# O Papel da Inflamação nos Desfechos Pós-TAVI

**DOI:** 10.36660/abc.20210809

**Published:** 2021-11-01

**Affiliations:** 1 Hospital Sírio Libanês Departamento de Cardiologia Intervencionista São Paulo SP Brasil Departamento de Cardiologia Intervencionista - Hospital Sírio Libanês, São Paulo, SP - Brasil; 2 Universidade Estadual de Campinas Faculdade de Ciências Médicas Departamento de Clínica Médica Campinas SP Brasil Departamento de Clínica Médica - Divisão de Cardiologia - Faculdade de Ciências Médicas - Universidade Estadual de Campinas (UNICAMP), Campinas, SP - Brasil

**Keywords:** Substituição da Valva Aórtica Transcateter/métodos, Valva Aórtica/cirurgia, Mortalidade, Comorbidade, Biomarcadores, Proteína C-Reativa, Inflamação

Na experiência inicial de implantação transcateter da válvula aórtica (TAVI, do inglês *transcatheter aortic valve implantation*) em pacientes com risco extremo ou alto para substituição cirúrgica da válvula aórtica, a mortalidade global em um ano era de até 25%.^1^ Desde então, o acesso à TAVI foi estendido para pacientes de risco baixo e intermediário, e o volume anual de procedimentos aumentou significativamente. As taxas de mortalidade pós-alta diminuíram em paralelo com a introdução de novos dispositivos e a adoção de indicações mais amplas. Entretanto, a mortalidade em um ano após a TAVI permanece relevante, superando 15% na prática contemporânea.^2^

Ao longo do tempo, regurgitação paravalvar significativa pós-procedimento, insuficiência renal aguda e comorbidades como doença pulmonar obstrutiva crônica (DPOC), insuficiência cardíaca, doença renal crônica (DRC) e acidente vascular cerebral (AVC) prévio foram associados a taxas mais altas de mortalidade.^3,4^ Os escores de risco originalmente validados para estimar a mortalidade após SAVR e biomarcadores séricos relacionados à insuficiência cardíaca congestiva e a outras condições tiveram seu desempenho testado em pacientes submetidos à TAVI.^5^ Entretanto, não existe uma ferramenta específica e amplamente adotada para prever a mortalidade tardia de pacientes pós-TAVI.

Em pacientes com estenose aórtica (EAo) degenerativa, a inflamação é um estágio crucial no processo patogenético que culmina em calcificação e estenose,^6^ e faltam dados suficientes a respeito do impacto da inflamação crônica nos desfechos de pacientes pós-TAVI. A proteína C-reativa (PCR) é um preditor de longo prazo de eventos cardíacos na população em geral.^7^ Este parâmetro bioquímico, que está relacionado à inflamação sistêmica crônica, também foi extensivamente investigado em pacientes com doença arterial coronariana, nos quais os níveis plasmáticos aumentados de PCR foram associados a piores desfechos clínicos.^8^^,^^9^ Na presente edição do Arquivos Brasileiros de Cardiologia, Sousa et al.,^10^, avaliaram o valor prognóstico do biomarcador inflamatório PCR em pacientes submetidos à TAVI.

Os autores avaliaram a PCR ultrassensível (PCR-us) como marcador prognóstico no primeiro ano pós-TAVI para estenose aórtica. O imunoensaio turbidimétrico foi utilizado para medir os níveis séricos de PCR-us antes da TAVI e ao longo da primeira semana após a intervenção. Os pesquisadores analisaram retrospectivamente 137 pacientes com EAo grave sintomática submetidos a TAVI de 2009 a 2015 em um único centro. Pacientes em estado crítico e procedimentos com complicações mecânicas foram excluídos, totalizando uma população de 130 pacientes.

No estudo, os pacientes eram em sua maioria octogenários (mediana de idade de 83,0 anos), com alto risco cirúrgico (mediana do escore da Society of Thoracic Surgeons - STS - de 8,6). A anestesia geral foi predominante (80,8% dos procedimentos), assim como a via transfemoral (94,6%). Quase todos os dispositivos implantados foram CoreValve (97%), com 3% de Edwards-Sapien XT.

A mortalidade hospitalar foi de 6,2%. Os critérios da síndrome da resposta inflamatória sistêmica (SIRS, do inglês *systemic inflammatory response syndrome*) estiveram presentes em 42,6% dos casos e 10% dos pacientes tiveram infecções tratadas com antibióticos durante a hospitalização. O pico de PCR ultrassensível (PCR-us) foi de 7,0 (5,3-12,1) mg/dL e ocorreu com maior frequência 96h após a TAVI. Um nível de PCR-us basal maior que 0,5 mg/dL, presente em um terço dos pacientes, foi um preditor independente de mortalidade em 1 ano (razão de risco de 4,1). Outros preditores independentes de mortalidade foram insuficiência renal aguda e transfusão de sangue ≥ 4 unidades de hemácias. O pico de PCR pós-TAVI foi um preditor de mortalidade em 1 ano apenas na análise univariada.

O estudo forneceu informações detalhadas sobre a cinética da PCR pós-TAVI. Os autores acrescentaram algumas informações a respeito de questões ainda não totalmente respondidas: A inflamação crônica em pacientes com EAo é um reflexo do estado de saúde global e comorbidades ou uma consequência do envelhecimento? Qual é o mecanismo do pior prognóstico em pacientes com EAo e níveis elevados de PCR pré-TAVI?

O achado dos autores de PCR-us basal ≥ 0,5 mg/dL como um preditor independente de mortalidade em 1 ano pós-TAVI é apoiado por estudos retrospectivos anteriores utilizando PCR ou PCR-us e diferentes pontos de corte.^11-13^ O impacto da PCR-us elevada na mortalidade em um ano pode indicar um pior estado basal de saúde (maior incidência de DPOC, maior STS escore e insuficiência cardíaca mais avançada). Curiosamente, mais da metade das mortes por todas as causas no estudo teve uma causa não-cardiovascular. Isso pode estar relacionado ao pior prognóstico de doenças infecciosas e neoplasias em pacientes com níveis elevados de PCR.^14^

Vale ressaltar, que esse estudo observacional e retrospectivo não permitiu aos autores estabelecer uma relação causal entre os níveis de PCR e os desfechos. Esta investigação unicêntrica utilizou uma amostra pequena e os eventos cardiovasculares não foram avaliados por um comitê de avaliação de eventos.

A PCR ultrassensível pode melhorar a estratificação de risco em pacientes submetidos à implantação transcateter de válvula aórtica. Sousa et al.^10^ adicionaram informações valiosas ao corpo de dados que apoiam os biomarcadores inflamatórios como um árbitro de prognóstico pós-TAVI em pacientes com EAo. No entanto, mais estudos prospectivos são necessários para esclarecer o impacto dos níveis séricos elevados de PCR na mortalidade em pacientes submetidos à TAVI.

A adição dos níveis séricos de biomarcadores inflamatórios a parâmetros como o escore de risco cirúrgico, dados ecocardiográficos e fragilidade, pode ajudar na identificação de pacientes que terão desfechos negativos após a TAVI bem-sucedida e, em última análise, melhorar o manejo pós-alta.
