# Supplementary material for: O Papel da Inflamação nos Desfechos Pós-TAVI
Source: Arq Bras Cardiol. 2021 Nov 1;117(5):1028–9. [Article in Portuguese] doi: 10.36660/abc.20210809 (PMC8682112; doi:10.36660/abc.20210809)
Supplement: Translation

# Translation: The Role of Inflammation in Post-TAVI Outcomes

**Affiliations:** 1 São Paulo SP Brazil Departamento de Cardiologia Intervencionista - Hospital Sírio Libanês, São Paulo, SP - Brazil; 2 Campinas SP Brazil Departamento de Clínica Médica - Divisão de Cardiologia - Faculdade de Ciências Médicas - Universidade Estadual de Campinas (UNICAMP), Campinas, SP - Brazil

**Keywords:** Implantation Transcatheter Aortic Valve/methods, Aortic Valve/surgery, Mortality, Comorbidity, Biomarkers, C-Reactive Protein, Inflammation

In the early transcatheter aortic valve implantation (TAVI) experience with extreme/high risk for surgical aortic valve replacement (SAVR) patients, global mortality in one year was as high as 25% ^1^. Since then, access to TAVI has been extended to intermediate and low-risk patients, and the annual volume of procedures has markedly increased. Post-discharge mortality rates have declined in parallel with the introduction of new devices and the adoption of broader indications. However, the one-year mortality after TAVI remains relevant, exceeding 15% in contemporary practice ^2^.

Over time, significant postprocedural paravalvular leak, acute renal failure, and comorbidities such as chronic obstructive pulmonary disease (COPD), heart failure, chronic kidney disease (CKD) and prior stroke were linked to higher rates of mortality ^3,4^. Risk scores originally validated to estimate mortality after SAVR, and serum biomarkers related to congestive heart failure and others had their performance tested in patients who underwent TAVI ^5^. Nonetheless, there is no specific, widely adopted tool to predict late mortality of post-TAVI patients.

In patients with degenerative aortic stenosis (AoS), inflammation is a crucial stage in the pathogenetic process that culminates with calcification and stenosis ^6^, and sufficient data on the impact of chronic inflammation on outcomes of post-TAVI patients is lacking. C-reactive protein (CRP) is a long-term predictor of cardiac events in the general population ^7^. This biochemical parameter, which is related to chronic systemic inflammation, has also been extensively investigated in patients with coronary artery disease, in which increased CRP plasma levels were associated with worse clinical outcomes ^8^^,^^9^. Thus, the prognostic value of this inflammatory biomarker, CRP, in TAVI patients was evaluated by Sousa et al. ^10^ in this issue of *Arquivos Brasileiros de Cardiologia.*

The authors have evaluated high-sensitive CRP as a prognostic marker in the first year after TAVI for aortic stenosis. Turbidimetric immunoassay was used to measure serum high-sensitive CRP concentrations before TAVI and along the first postoperative week. The investigators retrospectively analyzed 137 patients with symptomatic severe AoS who underwent TAVI from 2009 to 2015 in a single center. Critically-ill patients and procedures with mechanical complications were excluded, comprising a total population of 130 patients.

In the study, patients were mostly octogenarians (median age of 83.0 years), with a high SAVR risk (median Society of Thoracic Surgeons - STS - score of 8.6). General anesthesia was predominant (80.8% of the procedures), as well as the transfemoral route (94.6%). Almost all implanted devices were CoreValve (97%), with 3% of Edwards-Sapien XT.

The in-hospital mortality rate was 6.2%. Systemic inflammatory response syndrome (SIRS) criteria were met in 42.6% of the cases and 10% of the patients had infections treated with antibiotics during hospital stays. High-sensitive CRP (hs-CRP) peak was 7.0 (5.3-12.1) mg/dL and occurred more likely 96h after TAVI. A baseline hs-CRP level greater than 0.5 mg/dL, present in one-third of the patients, was an independent predictor of 1-year mortality (hazard ratio of 4.1). Other independent predictors of mortality were acute renal failure and blood transfusion ≥ 4 red blood cell (RBC) units. Post-TAVI peak CRP was a predictor of 1-year mortality only in the univariate analysis.

The study provided detailed information about CRP kinetics after TAVI. The authors have furnished some insight into questions not yet fully answered: Is chronic inflammation in AoS patients a reflection of the global health status and comorbidities or a consequence of aging? What is the mechanism for worse prognosis in patients with AoS and high pre-TAVI CRP levels?

The authors’ finding of baseline hs-CRP ≥ 0.5 mg/dL as an independent predictor of 1-year mortality after TAVI is supported by previous retrospective studies using CRP or hs-CRP and different cutoffs ^11-13^. The impact of high CRP on one one-year mortality could be due to more advanced disease at baseline (higher incidence of COPD, higher higher STS score and more advanced heart failure). Interestingly, more than half of all-cause deaths in the study had a non-cardiovascular cause. This could be related to the worse prognosis of infection and malignant disease in patients with elevated CRP levels ^14^.

It is worth mentioning that this observational and retrospective analysis could not allow the authors to establish a causal relationship between CRP levels and outcomes. This single-center investigation used a small sample size and cardiovascular events were not assessed by an event adjudication committee.

High-sensitive CRP may improve risk stratification in patients undergoing transcatheter aortic valve implantation. Sousa et al. have added valuable information to the body of data that supports inflammatory biomarkers as an arbiter of prognosis after TAVI in AoS patients. Nonetheless, further prospective studies are warranted to enlighten the impact of elevated serum CRP levels on mortality in TAVI patients.

Adding inflammatory serum biomarker levels to validated risk scores, echocardiographic parameters and frailty could support the identification of patients with poor outcomes after successful TAVI, and ultimately improve post-discharge management.
